# Strengths-based Nursing and Healthcare in maternities: rethinking practices and continuity of care^*^

**DOI:** 10.1590/1980-220X-REEUSP-2021-0597

**Published:** 2022-06-03

**Authors:** Otília Beatriz Maciel da Silva, Elizabeth Bernardino, Paula Encarnação

**Affiliations:** 1 Universidade Federal do Paraná, Curitiba, PR, Brazil.; 2 Universidade do Minho, Braga, Portugal.; 3 Pesquisadora UICISA-E, Escola de Enfermagem. Coimbra, Portugal.

**Keywords:** Nursing, Nurse Midwives, Women’s Health, Continuity of Patient Care, Holistic Nursing, Nursing Theory, Enfermagem, Enfermeiras obstétricas, Saúde da mulher, Continuidade da Assistência ao Paciente, Enfermagem Holística, Teoria de enfermagem, Enfermería, Enfermeras Obstetrices, Salud de la Mujer, Continuidad de la Atención al Paciente, Enfermería Holística, Teoría de Enfermería

## Abstract

**Objective:**

To identify elements of the Strengths-Based Nursing and Healthcare in the maternity nurses care practice in a perspective of continuity of care.

**Method:**

Qualitative exploratory-descriptive study. A focus group was used for data collection, seven meetings were held with 18 nurses between August 2019 and January 2020, starting from *a priori* categories: “problem-based nursing care” and “strengths-based nursing and healthcare*”.*

**Results:**

In the first category, nurses’ care is centered on problems identified in women; they keep a hierarchical relationship and a prescriptive posture based on a biomedical model. In the second category, care is focused on singularity, empowerment, self-determination, learning, collaborative partnership, and promotion of women’s health, based on a holistic nursing model.

**Conclusion:**

Although nurses use the biomedical model in their care practice, many of them already use the framework elements empirically. Applying this theoretical framework allows nurses to shift the focus of their attention from the disease to the person/family, promoting health and the continuity of care in a holistic way.

## RESUMO

**Objetivo:**

Identificar na prática assistencial dos enfermeiros da maternidade elementos do “*Strenghts-Based Nursing and Healthcare*”, em uma perspectiva de continuidade do cuidado.

**Método:**

Estudo exploratório-descritivo qualitativo. Utilizou grupo focal para coleta de dados, foram realizados sete encontros com 18 enfermeiros entre agosto de 2019 e janeiro de 2020, partindo-se de categorias a *priori*: “o cuidado de enfermagem baseado nos problemas” e “cuidado de enfermagem baseado no *Strenghts-Based Nursing and Healthcare”.*

**Resultados:**

Na primeira categoria, os enfermeiros cuidam centrados nos problemas identificados nas mulheres; mantêm relação hierárquica e postura prescritiva assente num modelo biomédico. Na segunda categoria, os cuidados centram-se na singularidade, empoderamento, autodeterminação, aprendizagem, parceria colaborativa e promoção da saúde da mulher, assente num modelo de enfermagem holístico.

**Conclusão:**

Apesar de o modelo biomédico ser utilizado na prática assistencial dos enfermeiros, muitos deles já utilizam empiricamente os elementos do referencial. Aplicar esse referencial teórico permite aos enfermeiros deslocar o foco de sua atenção da doença para a pessoa/família, promover a saúde e a continuidade dos cuidados de forma holística.

## RESUMEN

**Objetivo:**

Identificar en la práctica asistencial de los enfermeros de la maternidad elementos del “*Strenghts-Based Nursing and Healthcare*”, en una perspectiva de continuidad del cuidado.

**Método:**

Estudio exploratorio-descriptivo cualitativo. Se utilizó grupo focal para la recolección de datos, fueron realizados siete encuentros con 18 enfermeros entre agosto de 2019 y enero de 2020, iniciando con categorías a *priori*: “el cuidado de enfermería basado en los problemas y “cuidado de enfermería basado en el *Strenghts-Based Nursing and Healthcare”.*

**Resultados:**

En la primera categoría los enfermeros cuidan y se fundamentan en los problemas identificados en las mujeres; mantienen relación jerárquica y postura prescriptiva siguiendo un modelo biomédico. En la segunda categoría, los cuidados se concentran en la singularidad, empoderamiento, autodeterminación, aprendizaje, colaboración y promoción de la salud de la mujer, basada en un modelo de enfermería holístico.

**Conclusión:**

A pesar del modelo biomédico ser utilizado en la práctica asistencial de los enfermeros, muchos de ellos ya utilizan empíricamente los elementos del referencial. Aplicarlo permite a los enfermeros cambiar el enfoque de su atención de la enfermedad a la persona/familia, promoción de la salud y la continuidad de los cuidados de forma holística.

## INTRODUCTION

Obstetrics is constituted and supported by public policies considered the legal framework for this line of care, establishing health care flows and programs that strengthen the bond with women. The better the performance, the connectivity of care, the better the quality of care, the health outcomes for this user, as well as the cost-effectiveness benefits for the population and the health system^(1,2)^.

As a fundamental agent in this scenario, nurses are outstanding, especially obstetric nurses. These professionals follow a care model that seeks to rescue values such as woman’s protagonism, individuality, privacy, and autonomy, aiming at the promotion of healthy births, eliminating unnecessary interventions, and offering others that are proven to be beneficial^(3,4)^.

However, it is clear that these professionals have difficulty in changing their care practice still anchored in the biomedical model, in the technicality, and in the care plan based on the survey of problems and deficits. In the health system, this model of care is dominant and health workers are trained to identify problems and correct deficits^(5)^. Seeking a new perspective for obstetric nursing care, the assumptions of the Strengths-Based Nursing and Healthcare (SBNH) were chosen^(5)^. This perspective rescues values of professional care and can contribute to obstetric care, placing the woman and her family as active actors in the gestational process.

SBNH is a whole-person approach, focusing on what is working well, on what the person does best, and on the resources individuals have to help them better cope with health care challenges. It has values that lead nurses to the roots of care, focused on the individual identity and humanity, taking care of people and not just the diseases and problems presented^(5)^.

This study aims to identify the elements of the SBNH framework in the maternity nurses care practice, in a perspective of continuity of care.

## METHOD

### Design of Study

This is a qualitative exploratory-descriptive study.

### Population

Assistant and administrative nurses from a usual-risk maternity hospital, located in Curitiba, PR, Brazil, were invited to participate in the study.

### Selection Criteria

Assistant and administrative nurses with experience of at least one year working in the usual-risk maternity hospital and with availability to participate in focus group meetings, which were held between August 2019 and January 2020, were selected. Nurses that were absent due to vacation or sick leave were excluded.

### Data Collection

To present the theoretical framework Strengths-Based Care and Healthcare (SBNH) and provide reflection on its adoption in the professional practice of these nurses, the focus group was chosen as a data collection technique. The main investigator acted as moderator in these meetings. Seven focus group sessions were held, lasting 60 to 75 minutes. The themes developed in the meetings sought to identify whether nurses empirically adopted the SBNH framework in their practice of care in the maternity ward, from a perspective of continuity of care.

The focus group development was carried out taking the following steps: planning of actions to be developed in the group sessions; setting (meeting room preparation with posters presenting the values of SBNH and other elements of the theoretical framework); recruitment (invitation letters were delivered to all nurses in the institution, inviting them to participate in the study); group sessions (following the moments: opening of the session; presentation of the study proposal and the work method; establishment of the discussion; synthesis; session closure, and agreement of the new meeting), and evaluation (the study participants were invited to fill in an online questionnaire through the platform *Office Forms* ^®^ to evaluate the proposal developed in the focus group meetings and the possibility of applying the theoretical framework in the maternity professional practice^(6)^.

### Data Analysis and Treatment

The meetings were fully transcribed, with subsequent organization to improve the text and exclude vices of the language and repeated reports. Data were stored and organized in the software *MaxQDA* ^®^, analyzed according to the steps of Creswell’s theoretical methodological framework of content analysis^(7)^.

### Ethical Aspects

Following the rules of Resolution no. 466/12 of the National Health Council, which govern research involving human beings^(8)^, the project was submitted in 2018 to the Research Ethics Committee of the institution where the study was carried out, and after approval, in the same year, according to opinion n. 3.037.117 of November 25, 2018, data collection was started. The focus group meetings took place with the consent given by the participant and the signing of the Free and Informed Consent Form (FICF). To preserve anonymity, participants were named with the letter “E” followed by a sequential number (Ex. E1, E2, E3…).

## RESULTS

Eighteen nurses participated in the study, 16 women and two men, aged between 29 and 60 years, with an average time since end of under-graduation of ten years, and average time of work in the maternity hospital of nine years; thirteen workers were obstetrics specialists.

The content analysis of this study started from two *a priori categories*: “problem-based nursing care” and “SBNH-based nursing care”.

For the category “problem-based nursing care”, two subcategories were identified, the prescriptive posture and a hierarchical relationship with the woman, influenced by pre-established judgments. And for the category “SBNH- based nursing care”, SBNH elements were identified as subcategories: woman’s singularity; person-centered care; empowerment; self-determination; learning, preparation and timing; collaborative partnership; and health promotion. A model in transition is perceived, as there are elements that represent the biomedical model; however, elements that are in line with the category “SBNH-based nursing care” are also identified. This transition may have been stimulated by the humanization policies that have long anchored this work process.

**Chart 1.**
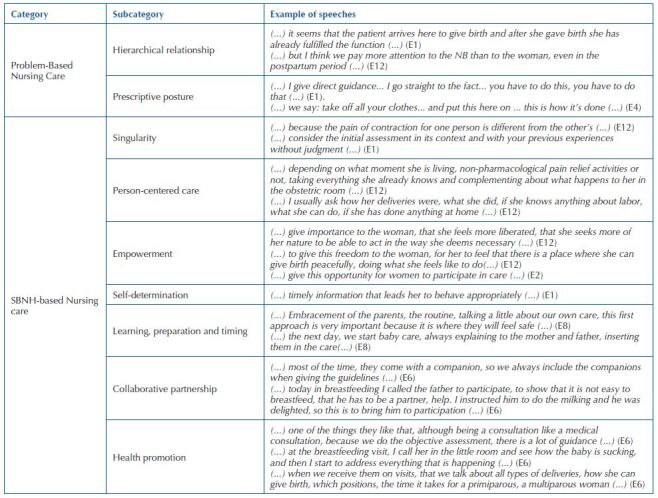
Categories and subcategories of elements of problem-based nursing care and elements of the SBNH-based care – Curitiba, PR, Brazil, 2021.

Chart 1 presents the categories, subcategories, and examples of the speeches.

## DISCUSSION

Deficits-based care is the dominant thinking in the health system, mainly due to the prevalence of the biomedical model, which has been developed over the years to help health professionals understand their patients’ clinical problems, reach an accurate diagnosis, and find the best treatment^(5,9,10,11)^.

In this model, the relationship between the professional and the patient takes place in a traditional hierarchical manner. The professional is the holder of knowledge and the patient assumes a passive role as the recipient of care. Differential diagnosis, clinical reasoning, and approaches based on the diagnosis of problems are the tools for decision making^(5)^.

Studies present assistance focused on meeting the pregnant woman’s health problem with little consideration regarding their doubts, wishes, feelings, and beliefs, and it is noticeable that health care in a humanized and welcoming way has not yet reached the level recommended by national and international guidelines^(12,13,14)^.

The prescriptive posture of nurses and the hierarchical relationship were examples of care based on deficits, in the reality studied. In obstetric care, the authoritarian structure of health professionals, specialists, legitimized over the years, provides a privileged position of power in the relationship with women, interfering with their autonomy and the care provided^(12–14)^.

One cannot deny that the advances made in this line of care have brought progress to maternal and fetal health. However, technological and therapeutic innovations in medical services, the relationship of professionals with parturients, and the way of conducting childbirth can still be considered factors that contribute to the dehumanization of care^(15)^. Remaining in the biomedical model can make care rigid to the point that weaknesses are not perceived and/or are considered common and adequate.

Despite the existing biomedical model in the maternity hospital of the study, it should be noted that SBNH elements are strongly present in nurses’ care practice in all care points of this institution, corroborating a care reality in transformation. These professionals aim at the creation of a bond with women. They consider their singularity, recognize and incorporate inner strengths and the family support network, use their professional competence and empathy for women, encourage their empowerment, and discover opportunities to help woman dealing with life events.

In this context, some elements of SBNH present in the care practice of maternity nurses were perceived, including the person’s singularity, person-centered care, empowerment, self-determination, learning, preparation and timing, collaborative partnership and health promotion.

Considering the person’s singularity is one of the values of the theoretical framework, SBNH identified in nurses’ practice in this study. This value provides the understanding that no two people are the same, because each individual has special qualities, a unique context, is influenced by the environment, and goes through unique experiences that define their personality^(5)^.

The role of the obstetrics nurses is to act encouraging the woman’s singularity, in her protagonism, in good practices, in the humanization of childbirth care, positioning themselves as mediators in the implementation of a new model of obstetric and neonatal care^(16)^.

Person-centered care refers to respectful care, in which the woman is able to be active in decisions about interventions related to her pregnancy, childbirth, and postpartum period. Her preferences, doubts, needs, and individual values shall be considered and contemplated in clinical decision-making, thus stimulating her empowerment^(17)^.

Empowerment has as its premise the belief that each person, family, and community has the resources, capabilities, skills, competencies, and potential to take responsibility for their own health, as well as to gain some mastery over their lives. Health professionals create conditions that allow people to acquire the necessary skills to promote their own empowerment, requiring emphasis on the inherent and acquired strengths present in individuals, families, and communities^(5)^. This principle is in line with the value of SBNH, self-determination, which provides the person with the right to choose and act according to their own thoughts, needs, and feelings, exercising their free will and making choices autonomously^(5)^.

The nurse is responsible for transmitting information and clarifying knowledge about pregnancy and childbirth. For the process of empowerment and self-determination, an attitude of communication, negotiation, and correlation between nurses and pregnant women is necessary, as well as the recognition of their potential^(18)^. This communication process is strongly linked to learning moments, also considered a value of the SBC (learning, preparation, and timing), being present in the professional practice of nurses in this study. Learning is defined as the acquisition of information, knowledge, and skills through experiences that lead the person to acquire new knowledge and to develop new skills, abilities and competences, to be able to adapt to different circumstances and to function in a changing world^(5)^. There are several factors affecting learning, among them, there are three essential conditions of the person: active involvement in this process, preparation, and timing of learning^(5)^.

Learning requires one to be an active participant, and this involvement requires attention to what is happening, to select what is relevant, to be able to store and create representations, and to have the ability to regulate, plan, and execute a variety of actions. For these reasons, it is essential that nurses assess the way the person learns, structuring activities and the learning environment to capitalize and develop new strengths^(5)^.

For these strategies to contribute to the pregnant woman’s learning, she has to be prepared. The preparation of a person for learning is a critical and essential factor for change, because learning and change are difficult to happen when there is no preparation. Nurses have to consider the individuals’ preparedness status and how they prepare for change. They need to look for signs of readiness and intervene to help them be ready for change^(5)^.

Learning is affected by timing, which requires an understanding of what will be changed and when the change is most likely to occur. Individuals are most receptive to change when they show openness to learning during transition periods, critical life events, and stressful experiences. Every new experience is an opportunity to learn about oneself, relationships, and how the world works^(5)^.

Pregnancy, childbirth, and the puerperium are largely involved in a continuous process of learning and care. For learning to take place, the woman’s individuality shall be respected, without judgments on her, care shall be centered on her, with learning being provided for through the respect for her time to understand, thus allowing the establishment of a collaborative partnership relationship.

The application of collaborative partnership, another value of SBNH, indicates that in the maternity nurses care practice there is a division of responsibilities among these professionals, women, and their families, providing and encouraging their autonomy, in line with the theoretical and legal precepts of this line of care. The person moves from a passive recipient of care to a partner in their own care. The collaborative partnership approach to care requires, among other things, a willingness on the part of the nurse to share power with the person and to work together on mutually agreed goals^(5)^.

The collaborative partnership contributes to the health promotion movement; when the objective is to carry out health education, one shall know how to look carefully at each one’s personal interests and try to work in general on their particularities. Health promotion, disease prevention, and self-care have as their premise the belief that people can change their health behaviors and that they have the power to change, requiring people to make the best choices related to life style, and a different mindset on the part of individuals, families, community, and government^(5,19,20)^.

This study showed that many nurses empirically use several of the elements of SBNH and that this use became more conscious as the discussions progressed. These elements are aligned with the concept of continuity of care, providing a window of opportunity for in-depth discussion and its application in these professionals’ care practice. It should be noted that nurses who use SBNH elements have some similar individual characteristics, such as a natural approach to the theoretical and legal framework and to good practices in the obstetric care line; an open mind for the reconstruction of the care model; and the use of empathy and competence in favor of women’s protagonism.

The questionnaire answered by nurses after the sensitization period allowed a reflection on the possibility of applying SBNH in the practice of these professionals, with the following factors being highlighted: knowledge as a motivator for changes in the care model and the scenario and context as an influencer for the application of this framework in professional practice.

## LIMITATIONS

As a limitation of this study, the application of the framework for a determined period in a single maternity hospital stands out.

## CONCLUSION

In this study, we sought to identify the elements of the theoretical framework SBNH in care practice of nurses in a usual-risk maternity hospital in the capital of Paraná.

SBNH is a philosophical approach that proposes a rescue in the nursing professional practice. This approach is in line with the values used by obstetric nurses in the care of pregnant and postpartum women. This work gave contributions related to practice, teaching, and research. The elements of SBNH were identified in the professional practice of most nurses, even with the routine and specific challenges experienced at the time. However, some professionals maintain the problem- based care model, which limits the application of this model.

Developing SBNH as theoretical support for practice is something innovative for Brazilian nursing. This study allowed knowing this framework, its application in obstetric care, motivation for its application in other realities, as well as stimulating nurses’ academic and continuing education, based on a theoretical framework that is feasible in professional practice.

## ASSOCIATE EDITOR

Maria Luiza Gonzalez Riesco
